# Prevalence of persistent microalbuminuria and associated factors among HIV infected children attending a Tertiary Hospital in Northern Tanzania: a cross sectional, analytical study

**DOI:** 10.11604/pamj.2015.20.251.5429

**Published:** 2015-03-16

**Authors:** Ignatus Kissima Mosten, Bernadus Carolus Hamel, Grace Damas Kinabo

**Affiliations:** 1 Kilimanjaro Christian Medical University College, Tumaini University Makumira P.O. Box 2240, Moshi, Tanzania; 2 Department of Paediatrics and Child Health, Kilimanjaro Christian Medical Centre (KCMC), P. O. Box 3010, Moshi, Tanzania

**Keywords:** Microalbuminuria, proteinuria, HIV, children, HIV associated nephropathy (HIVAN)

## Abstract

**Introduction:**

Human Immunodeficiency Virus (HIV) infection is a significant cause of paediatric morbidity and mortality especially in Sub-Saharan Africa. It affects the kidney by injuring the glomerular and tubular epithelial cells causing leakage of albumin in urine. Microalbuminuria is known to be an early indicator of kidney injury including HIVAN. The purpose of this study was to identify the prevalence and factors associated with microalbuminuria among HIV infected children receiving care and treatment at Kilimanjaro Christian Medical Centre (KCMC).

**Methods:**

We conducted a cross sectional hospital based analytical study at KCMC from December 2012 to April 2013. It involved children who are HIV infected attending child centred family care clinics (CCFCC). Patients’ demographic and clinical characteristics were extracted from the file; physical examination performed. Urine samples were analysed for by HemoCue Albumin 201 system analyzer. Statistical package for social sciences (SPSS) version 16.0 was be used to process and analyze the data.

**Results:**

Three hundred thirty HIV-infected children under 18 years were recruited during the study period. Mean age was 119.4 (5-218) months. Prevalence of microalbuminuria by using HemoCue Albumin 201 analyzer was 28.8% (n = 95). Presence of microalbuminuria was significantly associated with severity of HIV disease progression according to WHO disease stage (p = 0.0015) and CD4 count less than 350 cells/µL (p = 0.044).

**Conclusion:**

The study has shown that microalbuminuria is common in HIV infected children. Early screening and treatment of microalbuminuria is important to minimize the risk of developing end stage kidney disease. Children with advanced HIV disease and those with CD4 count less than 350cells/µL should be given priority for urinary albumin screening in a setting without routine screening for microalbuminuria.

## Introduction

Human Immunodeficiency Virus (HIV) infection is a significant cause of paediatric morbidity and mortality [1]. Almost 90% of all children living with HIV are in Sub-Saharan Africa [1]. In 2011, there were 1.9 million new infections, of which 390,000 were children under 15 years, while there were 2.1 million deaths, of which 250,000 were children, which was 20% fewer than in 2005 [1]. Tanzania has been hit by the AIDS pandemic although the current estimates are beginning to show a decline in prevalence from 5.7% in 2009 to the current 5.1%; with an estimated population of 43 million, it has about 2 million people living with HIV, 10% of whom are children below the age of 15 years [1, 2]. By the end of 2010, the percentage of adults and children with advanced HIV infection receiving antiretroviral therapy was 53%, consisting of 355,359 adults and 29,457 children [3]. Furthermore, the total number enrolled on care was 740,040, among them 681,795 being adults and 58,245 children. As the life span of HIV infected children improved due to antiretroviral (ARV) medications we are now seeing late complications of HIV infection like renal complications [4].

Studies have shown an association between HIV infection and development of renal diseases, both in adults and children [5–7]. This warrants a close follow up and monitoring of these children before they develop End Stage Renal Disease (ESRD) since renal diseases are indolent and need time for progression [8]. Chronic kidney diseases are not so common in children and the true prevalence is currently unknown, therefore their management is not getting enough attention [9]. Childhood human immunodeficiency virus-associated nephropathy (HIVAN) is defined by the presence of proteinuria associated with mesangial hyperplasia or global-focal segmental glomerulosclerosis and microcytic transformation of renal tubules [7]. Genetic susceptibility has being implicated in enhancing the risk of HIVAN development and it has been shown that black race is the most affected group, commonly presenting with focal segmental glomerulosclerosis [10, 11]. Linkage studies have found genetic susceptibility loci for developing microalbuminuria and HIVAN on chromosomes 3q, 10q and 18q [12]. A polymorphism in the gene for angiotensin II type 1 (AT1) receptor located at chromosome 3q21-25 has been found to be associated with both diabetic and non-diabetic associated nephropathy [13]. Studies have linked the presence of glomerulosclerosis and genetic variants in the MYH9 gene located in the long arm of chromosome 22 [10, 14, 15]. According to a report from the US Renal Data System, HIVAN had the strongest association with black race in all causes of renal failure among patients who were on maintenance dialysis [13–15].

If all HIV infected children are screened for HIVAN and appropriate management instituted, there is a possibility of preventing ESRD in these children [8]. Microalbuminuria defined as urinary albumin excretion between 30 to 300mg/day [16, 17], is known to be an early marker of HIVAN but it is often undetected because it remains asymptomatic [18, 19]. The prevalence of microalbuminuria varies from region to region and within regions, e.g. in the USA it ranges from 11% [20] to 34% [21] and Africa from 12% [22] to 72% [23].

In Tanzania, there is currently no established protocol for diagnosing, treating and following up HIV infected children at risk to develop HIVAN. Screening for microalbuminuria may allow for early identification of renal involvement in paediatric patients with HIV/AIDS infection. If identified at an early stage, interventions like antiretroviral (ARVs) and angiotensin converting enzyme (ACE) inhibitors can be put in place to slow down or halt renal disease progression. This study was carried to determine the prevalence of persistent microalbuminuria and its associated factors among HIV infected children attending a tertiary hospital in Northern Tanzania.

## Methods

This was a cross-sectional analytical hospital based study conducted between December 2012 and April 2013. The study was conducted at Kilimanjaro Christian Medical Center (KCMC), a university teaching hospital providing service to northern-central zones of Tanzania. A total of 460 HIV- infected children have been enrolled into care at the clinic from 2006 to 2013 and are actively attending, some of the children have been transferred to other hospitals. Study participants were recruited consecutively from the pool of HIV infected children up to the age of 17 years attending child centred family care clinic (CCFCC), the only clinic in the country currently providing HIV care to the family using the child as the entry point. During the clinic days caregivers and children were informed about the study by the principal investigator

Those on TB medication, with sickle cell disease and diabetes mellitus, and those using highly active antiretroviral therapy (HAART) containing tenofovir based combination were excluded from the study as they are confounders (however no child in this study was on tenofovir based regimen). Febrile children with a temperature of = 38°C, who tested positive for urinary tract infection and proteinuria by urine dipstick (Occidem Biotech, Middlesex UK LOT 110325), were also excluded.

The clinical examination findings were recorded in a pretested case report form including WHO clinical stage, weight, height, blood pressure and ART status. Blood was sent to the laboratory for CD4 percentage for the children less than 5 years and CD4 count for those aged 5 years and above whose most recent CD4 count was done more than 6 months ago with flow cytometry and expressed in cells per cubic millimeter by BD FACSCallibur- Becton Dickinson immunocytometry manufactured in San Jose, CA USA.

All eligible study participants had their urine tested using dipstick and Hemocue Albumin 201analyzer Angelhom Sweden. The Hemocue machine is quantitatively displaying urine albumin values from 5mg/L to 150mg/L, and displaying LLL and HHH for values below and above the cut-off readings respectively. For urine values which read HHH the dilution method was used to recalculate the exact value in mg/L. Microalbuminuria was defined as on the spot urine albumin 20-200 mg/L equivalent to 30-300mg/day (by the manufacturer leaflet) and the patient was counted to have persistent microalbuminuria when it was present in two consecutive urine samples collected one month apart.

Advanced HIV disease was defined as WHO clinical stage 3 and 4, while stage 1 and 2 was defined as nonadvanced. Immunosuppression was defined as CD4 count less than 350 cells/µL in children aged 5 years and above, and for those below age 5 years a CD4 percentage less than 25.

Results were analyzed using statistical package for social sciences (SPSS) version 16. Continuous variables such as age, duration on HAART, CD4 count and percentage were analyzed and expressed as means and standard deviations. Comparison of means was done by using the Student's t-test. Univariate followed by multivariate logistic regression analyses were performed using Pearson's correlation coefficients to determine the predictors of microalbuminuria. For categorical variables Chi-squared test was used. P value < 0.05 was set as significant for the tested variable. Ethical approval was obtained from Kilimanjaro Christian Medical University College Research and Ethics Review Committee (clearance certificate number 500). Those caregivers who agreed to participate signed the informed consent form. Written assent was also obtained from adolescents 10 years and older.

## Results

Three hundred and forty HIV-infected children aged up to 17 years were recruited during the study period. Ten children were excluded: four started anti-tuberculosis treatment and six were not available to provide the second urine specimen. Three hundred and thirty HIV infected children were included in the final analysis. There was a slight preponderance of male children n = 182 (55.2%). Mean (SD, range) age was 119.4 (48.7, 5 to 218) months. Majority of children (n = 288, 87.3%) were 60 months or older. Three hundred (90.9%) of the HIV infected children were on HAART, while 238 (79.3%) children were on first line regimens and 62 (20.7%) on second line regimen. Ninety two (27.9%) were on cotrimoxazole prophylaxis.

Prevalence of microalbuminuria was 28.8% (n = 95). The mean age of the children with microalbuminuria was 123.1±52.5 months. The percentage of children with microalbuminuria and aged less than 60 months was 33.3.0% (n = 14) while in those aged at least 60 months it was 28.1% (n= 81) which was not statistically different. There was a trend towards significance for the duration of HAART, as those children on HAART for more than 5 years were less likely to have microalbuminuria than those on HAART for less than five years.

Table 1 shows the distribution of clinical characteristics of children. Majority of the study subjects (229, 69.4%) had advanced HIV diseases defined as WHO clinical stage III n = 150 (45.5%) and stage IV n = 87 (26.4%). CD4 cell count ranged from 19 to 4732 cells/µL. The median (IQR) absolute CD4 cell count was 699 (474 to 1046) cells/µL. Median (IQR) CD4 cell count for the children 60 months and older was 679 (450 to 928) while for under five children the corresponding figures were 1155 (735 to 1906). For the children under five years, mean (SD, range) CD4 percentage was 30.4 (8.9, 13 to 49).

**Table 1:** Clinical characteristics of the study population (n = 330)

Variable	N (%)
**WHO stage:**	
Stage I	28 (8.4)
Stage II	75 (22.7)
Stage III	150 (45.5)
Stage IV	87 (26.4)
Median (IQR) CD4 count (cells/µL) (n = 330):	699 (474 to1046)
Median (IQR) CD4 count for children <60 months (n = 42):	1155 (735 to 1906)
Median (IQR) CD4 count for children ≥60 months (n = 288):	679 (450 to 928)
Mean (±SD, range) CD4 percentage (n= 42) under 60 months	30.4 (±8.9, 13 to 49)
Mean (±SD, range) systolic blood pressure (BP)(mmHg) (n = 330):	92 (±9, 70 to 120)
Mean (±SD, range) systolic BP for children <60 months (mmHg):(n = 42):	87 (±6, 70 to 100)
Mean (±SD, range) systolic BP for children ≥60 months (mmHg) (n = 288):	93 (±9, 70 to 120)
Mean (±SD, range) diastolic blood pressure (mmHg) (n = 330):	59 (±8, 40 to 80)
Mean (±SD, range) diastolic BP for children <60 months (mmHg) (n = 42):	54 (±7, 40 to 60)
Mean (±SD, range) diastolic BP for children ≥60 months (mmHg) (n = 288):	60 (±8, 40 to 80)
Mean (±SD, range) Body Mass Index (BMI) for children ≥60 months (n = 288):	16.3 (±2.5, 11.7 to 29.1)

Mean (SD) systolic and diastolic blood pressures for all children were in the normal range (92±9 and 59±8 mmHg respectively). More than three quarters of children 282 (85.5%) had normal nutritional status while only 6 (1.8%) were severely malnourished. Mean body mass index (BMI) was 16.3±2.5 kg/m^2^ (for children at least 60 months old) and mean z-score for weight-for-height (WHZ) for under 60 months was -0.2SD (Table 1).

Of 330 children under 18 years, 300 (90.9%) had started ARV use. Duration of ARV use ranged from 1 to 172 months. Median (IQR) duration was 48 (25 to 72) months. One hundred and ninety four (58.6%) children had been on HAART for less than five years. Of 300 children on ARV, 62 (20.7%) changed to second line ART. Of 62 children who changed to second line ART, mean (SD, range) time lapse before change was 36.3 (21.7, 8 to 108) months.

Characteristics of HIV-infected children with and without microalbuminuria are shown in Table 2. Presence of microalbuminuria was significantly associated with severity of HIV disease progression according to WHO disease stage (p = 0.0015), CD4 count less than 350 cells/µL (p= 0.044) and small BMI (p = 0.027). Microalbuminuria was detected more in HIV-infected children who had been on ART for a shorter period compared to longer period (mean duration on ART 44.1 vs. 51.2 months), however the difference between the groups did not reach statistical significance (p = 0.076). The majority n = 57 (73%) of HIV infected children with microalbuminuria had been on HAART for less than 5 years and they were not statistically different from the group without microalbuminuria. Other patient characteristics did not significantly relate to presence or absence of microalbuminuria (p > 0.05).

**Table 2:** Relationship between characteristics of the study population and microalbuminuria (n = 95)

VARIABLE*	Microalbuminuria		p-value	Odds Ratio 95% CI
	+ve (n = 95)	-ve (n = 235)		
	No. (%)	No. (%)		
**Sex:**				
Male	47(25.8)	135(74.2)		
Female	48(32.4)	100(67.6)	0.187	0.725(0.450-1.170)
**Age (months):**				
Under 60	14 (33.3)	28 (66.7)		
At least 60	81(28.1)	207(71.9)	0.486	1.278(0.640-2.550)
**WHO clinical stage**				
Stage I + II	15 (16.0)	78 (84.0)		
Stage III + IV	80 (33.8)	157 (66.2)	0.0015	0.524(0.204-0.698)
**HAART use status:**				
On HAART	87(29.0)	213 (71.0)		
Not on HAART	8 (26.7)	22 (73.3)	0.788	1.123(0.482-2.619)
**Duration on HAART**				
Less than 60 months	57(29.4)	137(70.6)		
At least 60 months	21(19.8)	85(80.2)	0.070	1.643(0.954- 2.974)
**HAART regimen:**				
1^st^ Line	65(27.3)	173(72.7)		
2^nd^ Line	22(35.5)	40(64.5)	0.207	0.683(0.377-1.236)
**CD4 count category**				
CD4 count < 350 cells/µL	21(40.4)	31 (59.6)		
CD4 count ≥ 350 cells/µL	74(26.6)	225(73.4)	0.044	1.868(1.010-3.452)
**‡CD4% category (age less than 60 months)**				
CD4 percent ≤ 25	2(22.2)	7(77.8)		
CD4 percent > 25	12(36.4)	21(63.6)	0.425	(0.045-3.290)

* Chi square test

‡ Fisher exact test

Backward stepwise logistic regression, including HIV disease progression category by WHO clinical stage (p= 0.0015), duration of HAART category of less or more than 60 months (p= 0.073), CD4 count category less or more than 350 cells per microlitre (p= 0.044) showed that HIV disease category and CD4 count category remained significantly associated with presence of microalbuminuria (Table 3).

**Table 3:** Logistic regression model of predictors of microalbuminuria

VARIABLE	B	S.E.	Wald	df	Sign.(p-value)
WHO clinical stage	-0.492	0.160	5.516	1	0.0015
Nutritional status	0.323	1.592	0.041	1	0.893
Duration on HAART	-0.674	0.312	4.659	1	0.060
HAART regimen	0.486	0.336	2.099	1	0.147
CD4 count category	-0.572	0.337	2.871	1	0.044

## Discussion

The prevalence of microalbuminuria in HIV infected children in our studywas 28.8%. The factors which were significantly associated with it were low CD4 count and advanced HIV disease. The nutritional status and antiretroviral therapy (ART) duration showed trends towards significant in association with the presence of microalbuminuria. The prevalence findings were similar to other studies conducted in USA [19–21], Europe and Africa which found a high prevalence of microalbuminuria in HIV infected children and adults [22–26].

However, our findings were different from the study conducted by Uzoma and colleagues in Nigeria who found zero prevalence of microalbuminuria [27]. This difference could probably be explained by the differences in characteristics of the study participants and methodology. Where as we studied subjects who were on HAART, the majority of whom had also advanced HIV disease, in contrast the majority of the subjects in Uzoma's study had non-advanced HIV disease. In our study we used the hemocue albumin analyzer which gives exact values of urine albumin in contrast to the micral strips used by Uzoma et al. which is semiquatitative [28]. HIV infected children in our study with absolute CD4 counts of more than 350/µL were less likely to present with microalbuminuria, while CD4 percentage did not influence the occurrence of microalbuminuria. This inverse relationship between CD4 count and presence of microalbuminuria is explained by the fact that as CD4 count declines the viral load increases and this escalates the possibility of renal infection by HIV [29]. Advanced HIV disease was found in this study to be significantly associated with microalbuminuria in children. Similar findings have been shown by other authors [24–26]. This can be explained by the fact that as the disease advances there is profound immunity suppression favouring unchecked viral replication and hence kidney involvement.

Using HAART was not influencing the occurrence of microalbuminuria. This was also observed by other researchers [30–34]. Apparently kidneys may act as reservoir for HIV and even at suppressed viral load, HIV can still induce kidney damage. It can also be explained by the presence of other intrinsic factors like genetic predisposition shown to be associated with the occurrence of kidney diseases but not assessed in this study [10–14]. We screened the presence of urine albumin in more than one urine sample taken at least one month apart to define microalbuminuria. This helped to exclude the possibility of transient microalbuminuria which is common in young age.

There were some limitations. We did not limit physical exercise 24 hour before urine collection and this could have had an effect on microalbuminuria especially in lean adolescents. Viral load was not tested in all children and therefore it was not possible to establish the association between viral load and the presence of microalbuminuria. Renal biopsy was not preformed and therefore in the children with microalbuminuria it was difficult to ascertain those who had already developed HIVAN.

## Conclusion

This urinary screening study has shown that microalbuminuria in HIV infected children is common. HAART use did not influence the presence of microalbuminuria in HIV infected children, but presence of advanced HIV disease and low CD4 count are significant predictors of microalbuminuria.

## References

[1] UNAIDS (2012). Report on the Global AIDS Epidemic. http://www.unaids.org/media/unaids/content/epidemiology/2012/gr2012/201.

[2] Tanzania HIV/AIDS and Malaria Indicator Survey (THMIS) (2011-2012). http://www.tacaids.go.tz/component/docman/doc_details/73-third-tanzania-hiv-and-malaria-indic….

[3] NACP: Epidemiology of HIV/AIDS (2012). NATIONAL GUIDELINES FOR THE MANAGEMENT OF HIV AND AIDS.

[4] TACAIDS:United Republic of Tanzania (2012). COUNTRY PROGRESS REPORT: Part A: Tanzania Mainland. http://www.unaids.org/en/dataanalysis/knowyourresponse/countryprogressreports/2012countrie….

[5] Conaldi PG, Biancone L, Botelli A, Wade-Evans A, Racusen LC, Boccelino M, Orland V, Serra C, Camussi G, Toniolo A (1998). HIV-1 kills renal tubular epithelial cells in vitro by triggering an apoptotic pathway involving caspase activation and Fas upregulation. J Clin Invest..

[6] Bruggeman L, Bark C, Kalayjian RC (2009). HIV and the Kidney. Curr Infect Dis Rep..

[7] Husain M, Gusella GL, Klotman ME, Gelman IH, Ross MD, Schwartz EJ, Cara A, Klotman PE (2002). HIV-1 Nef Induces Proliferation and Anchorage-Independent Growth in Podocytes. J Am Soc Nephrol..

[8] Ray PE, Hu CA (2011). Advances in our understanding of the pathogenesis of HIV-1 associated nephropathy in children. Future Virol..

[9] Kiryluk K, Martino J, Gharavi AG (2007). Genetic Susceptibility, HIV Infection, and the Kidney. Clin J Am Soc Nephrol. Clin J Am Soc Nephrol..

[10] Satko SG, Freedman BI, Moossavi S (2005). Genetic factors in end-stage renal disease. Kidney international.

[11] Freedman BI, Murea M (2010). Potential effects of MYH9-associated nephropathy on dialysis and kidney transplant outcomes. Semin Dial..

[12] Genovese G, Friedman DJ, Ross MD, Lecordier L, Uzureau P, Freedman BI, Bowden DW, Langefeld CD, Oleksyk TK, knob ALU, Bernhardy AJ, Hicks PJ, Nelson GW, Vanhollebeke B, Winkler CA, Kopp JB, Pays E, Pollak MR (2010). Association of Trypanolytic ApoL1 Variants with Kidney Disease in African Americans. Science.

[13] Quaggin SE (2009). Genetic susceptibility to HIV-associated nephropathy. J Clin Invest..

[14] Chow KM, Wong TYH, Li PK (2005). Genetics of common progressive renal disease. Kidney international. Supplement.

[15] Kopp JB, Winkler CA, Nelson GW (2010). MYH9 Genetic Variants Associated With Glomerular Disease: What Is the Role of Genetic Testing?. Semin Nephrol..

[16] Koroshi A (2007). Microalbuminuria, is it so important?. Hippokratia..

[17] De Zeeuw D (2004). Albuminuria, not only a cardiovascular/renal risk marker, but also a target for treatment?. Kidney international..

[18] Choi AI, O'Hare AM, Rodriguez R (2007). Update on HIV-associated Nephropathy. Nephrology Rounds.

[19] Szczech LA, Grunfeld C, Schezer R, Canchola JA, van der Horst C, Sidney S, Wohl D, Shlipak MG (2007). Microalbuminuria in HIV infection. AIDS..

[20] Szczech LA, Hoover DR, Feldman JG, Cohen MH, Gange SJ, Gooze L, Rubin NR, Young MA, Cai X, Shi Q, Gao W, Anastos K (2004). Association between Renal Disease and Outcomes among HIV-Infected Women Receiving or Not Receiving Antiretroviral Therapy. HIV/AIDS CID..

[21] Kim PS, Woods C, Dutcher L, Georgoff P, Rosenberg A, Mican JAM, Kopp JB, Smith MA, Hadigan C (2011). Increased Prevalence of Albuminuria in HIV-Infected Adults with Diabetes. PLos One..

[22] Han TM, Naicker S, Ramdial PK, Assounga GK (2006). A cross-sectional study of HIV-seropositive patients with varying degrees of proteinuria in South Africa. Kidney International..

[23] Fabian J, Naicker S, Venter W, Baker L, Naidoo S, Paget G, Wadee S (2009). Urinary screening abnormalities in antiretroviral-naive HIV-infected outpatients and implications for management- A single –center study in South Africa. Ethn Dis..

[24] Eke FU, Anochie IC, Okpere AN, Eneh AU, Ugwu RN, Ejilemele AA, Ugboma HU (2010). Microalbuminuria in children with human immunodeficiency(HIV) infection in Port Harcourt, Nigeria. Nigeria J Med..

[25] Msango L, Downs JA, Kalluvya SE, Kidenya BR, Kabangila R, Johnson WD, Fitzgerald DW, Peck RN (2011). Renal dysfunction among HIV-infected patients starting antiretroviral therapy. AIDS..

[26] Fredrick F, Ruggajo P, Maro E, Iversen BM, Basu G (2012). Renal manifestation and associated factors among HIV infected children at Muhimbili National Hospital, Dar-es- Salaam, Tanzania. BMC Infectious Diseases..

[27] Uzoma BE, Uchena OH, Nnaemeka IA, Tagbo O (2012). Screening for Microalbuminuria in HIV-Positive Children in Enugu. International Journal of Nephrology..

[28] Parikh CR, Fischer MJ, Estacio R, Schrier RW (2004). Rapid microalbuminuria screening in type 2 diabetes mellitus: simplified approach with Micral test strips and specific gravity. Nephrol Dial Transplant..

[29] Tindyebwa D, Kayita J, Musoke P, Eley B, Nduati R, Coovadia H, Bobart R, Mbori-Ngacha D, Kiefer MP, African Network for the Care of Children affected by AIDS (ANECCA) (2004). Epidemiology, Pathogenesis and Natural History. Handbook on Paediatric AIDS in Africa.

[30] Chaparro AI, Mitchell CD, Abitbol CL, Wilkinson JD, Baldarrago G, Lopez E, Zilleruelo G (2008). Proteinuria in Children Infected with the Human Immunodeficiency Virus. Pediatrics..

[31] Ekulu PM, Nseka NM, Aloni MN, Gini JL, Makulo JR, Lepira FB, Sumaili EK, Mafuta EM, Nsibu CN, Shiku JD (2012). Prevalence of proteinuria and its association with HIV / AIDS in Congolese children living in Kinshasa, Democratic Republic of Congo [abstract]. Nephrol Ther..

[32] Ramezani A, Mohraz M, Banifazl M, Jam S, Gachkar L, Yaghmaie F, Eslamifar A, Zadsar M, Kalantar N, Nemati K, Haghighi M, Rezaie M, Aghakhani A (2008). Frequency and associated factors of proteinuria in Iranian HIV-positive patients. International Journal of Infectious Diseases..

[33] Iduoriyekemwen NJ, Sadoh WE, Sadoh AE (2013). Prevalence of Renal Disease in nigerian Children Infected with the Human Immunodeficiency Virus and on Highly Active Anti-retroviral Therapy. Saudi J Kidney Dis Transpl..

[34] Elewa U, Sandri AM, Rizza SA, Fervenza FC (2011). Treatment of HIV-associated nephropathies. Nephron Clinical practice..

